# Make Gestures to Learn: Reproducing Gestures Improves the Learning of Anatomical Knowledge More than Just Seeing Gestures

**DOI:** 10.3389/fpsyg.2017.01689

**Published:** 2017-10-05

**Authors:** Mélaine Cherdieu, Olivier Palombi, Silvain Gerber, Jocelyne Troccaz, Amélie Rochet-Capellan

**Affiliations:** ^1^GIPSA-Lab, Université Grenoble Alpes, CNRS, Grenoble INP, Grenoble, France; ^2^LADAF, University Hospital, Université Grenoble Alpes, Grenoble, France; ^3^LJK, Université Grenoble Alpes, CNRS, Grenoble INP, Grenoble, France; ^4^TIMC-IMAG, Université Grenoble Alpes, CNRS, Grenoble INP, Grenoble, France

**Keywords:** manual gesture, learning and memory, embodied cognition, anatomy, long-term memory

## Abstract

Manual gestures can facilitate problem solving but also language or conceptual learning. Both seeing and making the gestures during learning seem to be beneficial. However, the stronger activation of the motor system in the second case should provide supplementary cues to consolidate and re-enact the mental traces created during learning. We tested this hypothesis in the context of anatomy learning by naïve adult participants. Anatomy is a challenging topic to learn and is of specific interest for research on embodied learning, as the learning content can be directly linked to learners' body. Two groups of participants were asked to look at a video lecture on the forearm anatomy. The video included a model making gestures related to the content of the lecture. Both groups see the gestures but only one also imitate the model. Tests of knowledge were run just after learning and few days later. The results revealed that imitating gestures improves the recall of structures names and their localization on a diagram. This effect was however significant only in long-term assessments. This suggests that: (1) the integration of motor actions and knowledge may require sleep; (2) a specific activation of the motor system during learning may improve the consolidation and/or the retrieval of memories.

## Introduction

The movements we make do not only act on the outer world but they also shape our cognitive abilities. A broad range of neurological and behavioral evidences suggest that the motor system is involved in perception, decision making, lexical representation, memory, and emotional processes (Wexler et al., 1998; Glenberg and Kaschak, 2002; Tyler et al., 2003; Barsalou, 2008; Casasanto and Dijkstra, 2010). Among the various body actions shown to influence our cognitive abilities, research paid particular attention to manual gestures, due to their multi-faceted representative function and their special relationship to language (McNeill, 1992; Goldin-Meadow and Alibali, 2013). Manual gestures are commonly and spontaneously used by speakers, learners and teachers. They were shown to support language learning on receptive and expressive sides, but conceptual learning and problem solving as well (Kontra et al., 2012; Novack and Goldin-Meadow, 2015). Experimental results also indicate that when children or adults gesture during learning they outperform participants who do not. Embodied and active learning is therefore a major focus, at the crossroad of cognitive and education sciences, but it is mainly investigated with children so far in regards to educational aspects (Kelly et al., 2008; Kontra et al., 2012; Novack and Goldin-Meadow, 2015). The role of gesturing in learning might depend on the topic of interest and population. In this paper, we evaluate the beneficial aspect of gesturing in anatomy learning in naïve adults. Anatomy is a challenging topic for students (Sugand et al., 2010; Zumwalt et al., 2010), involving visuo-spatial skills, mental imagery, as well as lexical memories. Making gestures to learn anatomy is also a specific case of embodied active learning, in which explicit knowledge on the body is developed throughout the implication of the body. Our experimental work compares anatomy learning of the forearm in controlled situation when seeing vs. seeing and imitating gestures related to the educational content.

### Background: the embodied cognition framework

Sensory-motor experiences and body movements, are a growing focus of researches in various fields of cognitive sciences. These researches have been developed in conjunction with the theoretical framework of “embodied cognition” (cf. Varela et al., 1993; Clark, 1999; Wilson, 2002; Barsalou, 2008; Borghi and Pecher, 2011). Nowadays, research on the implication of sensory-motor experiences in cognitive functions is clearly connected to and/or motivated by embodied cognition (Hostetter and Alibali, 2008; Ionescu and Vasc, 2014). This is also the case of the present paper. According to the embodied cognition framework, mental functions are grounded in sensory-motor processes linked to specific situations (“grounded cognition,” see Barsalou, 2008). Therefore, cognitive representations are not amodal but rather “grounded” in the sensory-motor processes involved in their construction. In knowledge acquisition, as in all experiences, “the brain captures states across the modalities and integrates them with a multimodal representation stored in memory” (Barsalou, 2008, p. 618). In this framework, cognitive processes rely on simulation mechanisms that could be defined as “the reenactment of perceptual, motor, and introspective states acquired during experience with the world, body, and mind” (Barsalou, 2008, p. 619). As specific cases of sensory-motor experiences, and due to their powerful representational and deictic functions, manual gestures are the core interest of a variety of studies focused for instance on the role of gestures in language, conceptual learning, problem solving or spatial representations. Different dimensions have been proposed to classify communicative gestures based on the analysis of narrative situations (Kendon, 1980; McNeill, 1992; Colletta et al., 2009). Among these gestures, the current study mostly focuses on iconic (“refers to a concrete event, object, or action that is also referred to in speech at the same time,” McNeill, 1992, p. 77) and deictic gestures (point to something, someone, somewhere…). These gestures were inspired by those made by a professor to explain the main notions in a lecture on forearm anatomy.

### Seeing gestures

A major human activity that clearly involves gestures is communication. In a daily basis, people are spontaneously gesturing in order to communicate. These gestures can mark the rhythm of the discourse, strengthen the message or provide additional information to the listener. In a recent meta-analysis, Hostetter (2011) highlighted the importance of gestures in receptive communication. Seeing speakers' gestures helps listeners to understand the spoken content of the message. This effect is more important when speakers' gestures provide supplemental information rather than redundant information to the spoken message, and it is especially relevant for children. These results suggest that gestures could represent “a second channel that makes successful comprehension more likely” (Goldin-Meadow and Alibali, 2013, p. 261). Gestures are thus seen as a facet of human language (McNeill, 1981; Kelly et al., 2008). The effect of gestures on language comprehension however depends on the information contained in the gestures (Goldin-Meadow and Alibali, 2013), and on the complexity of the spoken message (McNeill et al., 2000). Besides, the augmentation of a spoken message by gestures has a beneficial effect in education.

During class, teachers spontaneously gesture when they have to explain mathematical concepts to children (Goldin-Meadow et al., 1999) and several studies have shown that seeing the teacher's gestures during learning is beneficial to the learner. When children see gestures while they learn to solve problems in mathematics, they improve more between pre-test and post-test than when they receive oral instructions only (Cook et al., 2013). This has also been observed for concepts learning such as the concept of conservation (comparing the same amount of water in two different glasses, see Church et al., 2004; Ping and Goldin-Meadow, 2008) or symmetry (Valenzeno et al., 2003). Interestingly, Singer and Goldin-Meadow (2005) found that seeing gestures during a lesson of mathematics improves performance 24 h after the lesson and tends to facilitate the transfer to other problems. Teachers' gestures can help children to understand and integrate instructions, which can explain their positive effect on long-term performances. Here again, in order to be helpful, gestures should convey supplemental information to verbal content when children learn mathematics, they only improved when the teachers' gestures bring a different way to approach the problem than the teacher's spoken message. These results highlight the crucial role of gestures in language comprehension and in different types of conceptual learning. However, if seeing gestures is important, doing gestures also has a major impact in cognitive activities.

### Doing gestures

Gestures appear very early in the development of expressive communication, even before the first words. For example, children can point objects before they can name them. Not only gestures precede oral language, they seem related to it over the course of development: the early use of gestures in communication can predict some aspects of speech acquisition. In a longitudinal study, Iverson and Goldin-Meadow (2005) showed that the spoken lexical items developed by children around 24 months are tied to the gestures they have made during their early communicative activities, around 10 months. Early gestures do not only predict the lexical items, they also predict the vocabulary range. Thereby, gestures made by 14 months children predict their vocabulary size at 42 months (Rowe and Goldin-Meadow, 2009). Children also use gestures to organize their thoughts and solve problems. For instance, in Breckinridge Church and Goldin-Meadow (1986), children had to explain their understanding of the concept of conservation while judging the same amount of water poured into two glasses with different shapes. When they explained their reasoning to solve the problem, some of them spontaneously produced gestures matching their reasoning while others produced gestures mismatching their reasoning. The latters were more likely to understand their mistakes, and better improve their performance than the formers. The role of making gestures in the organization of thought in children has also been shown for mathematical problem solving (Goldin-Meadow and Beilock, 2010; Alibali and Nathan, 2012), moral reasoning (Beaudoin-Ryan and Goldin-Meadow, 2014) and text learning (Cutica et al., 2014). Besides supporting language production, learning, and reasoning, gestures are more generally involved in knowledge access. In a recent study, Gunderson et al. (2015) reported that when children gestured during number-knowledge tasks (tasks involving the evaluation of set sizes), they could have access to knowledge not included in their spoken message, that is to say “numbers above their knower-level” (Gunderson et al., 2015). In the same way, tracing during geometry learning enhance the children' knowledge, who then perform better in evaluation tests (Hu et al., 2015).

Gestures therefore appear as a supplemental dimension to learning, helping children who gesture to outperform children who don't gesture. This influence of gestures on thought, problem solving and knowledge access is yet not restricted to children. Rauscher et al. (1996) have shown that gesturing while speaking could facilitate access to the mental lexicon in spontaneous speech produced by undergraduate students. The positive influence of gestures was also assessed in learning new words, throughout second language learning: adults can learn a second language easier if they gesture during learning (McCafferty, 2004; Gullberg, 2006).

As observed in children, gestures are also an important part of the organization of thought and reasoning in adults. In a recent study (Trofatter et al., 2014), a group of students had to solve the Tour of Hanoi puzzle. This puzzle is used in neuropsychology to assess planning and reasoning. The students had to solve the problem once and then had to explain their reasoning. The students who gestured during their explanation showed a greater improvement when they solved the Tower of Hanoi again than those who did not make gestures. Gestures are quite important in learning, reasoning and language and should surely be taken into account in education for children as well as for adults (Ionescu and Vasc, 2014). These previous studies have recently inspired researches about the role of gestures in anatomy learning.

### The specific case of anatomy learning

Anatomy learning is particularly challenging for medical and paramedical students. Functional anatomy requires recalling the names, locations and functions of the different structures of the human body. Various studies investigated new teaching methods inspired by embodied cognition in order to facilitate anatomy learning. Therefore, painting anatomical structures on the learner's body (McMenamin, 2008) or drawings them on t-shirts (Skinder-Meredith, 2010) can have a positive effect on student's motivation and consequently possibly enhance performances (for a review see Sugand et al., 2010). However, most of these studies did not objectively investigate the effect of these methods on the performance in anatomy tests.

The potential role of gestures in anatomy learning is particularly interesting because it is about learning knowledge on the body throughout the body. Intuitively we can think that gestures will improve the memorization of anatomical structures and the understanding of their implication in body functions. This point has been developed in some studies using gestures in different ways. Oh et al. (2011) described what they called “digit anatomy”: students had to reproduce several fingers configuration to learn the anatomy of the heart. In this case, the fingers represented vessels, bones, and muscles. A subjective evaluation of the technique indicated that students found the method helpful to memorize the structures and to understand the functions.

Pointing and tracing gestures were also tested as supports of anatomy learning in MacKen and Ginns (2014). During the learning of the structures and functions of the human heart, some students were told to do pointing and tracing gestures on diagrams, while others were told not to use their hands (they had to sit on their hands to control that they would not make any gestures). Students who gestured during learning had better performance than those who did not, both for the terminology test (“knowledge of specific definitions and labels”) and the comprehension test (understanding of the structures and functions of the heart; MacKen and Ginns, 2014, p. 598). This result suggests that pointing and tracing gestures can improve the learning of heart anatomy.

These previous studies investigated the role of different types of gestures in anatomy learning of internal structures, in particular the heart. Others authors introduced the possibility to make movements to learn information about the structures and the functions involved in these particular movements. Therefore, Dickson and Stephens (2015) investigated if making facial expressions could improve the learning of cranial nerves involved in these expressions. Students were taught a lesson about these cranial nerves without doing any gesture (Didactic lecture) and then attended to the same lecture when asked to do facial expressions illustrating the functions of the different nerves in facial movements (Miming lecture). A pre-test and a post-test of knowledge were administered before and after each lecture. When comparing the results at pre-tests and post-tests the authors observed a greater improvement for the Miming condition as compared with the Didactic condition. It is however unclear if this improvement is related to the implication of movements or if it is only related to the fact that the lesson was received twice. Movements were also done collectively without any control of students' behavior. The study was acknowledged as a pilot study.

Based on these previous researches in cognitive sciences and education, our aim was to provide further evidences for the role of making gestures in the acquisition of new knowledge by adult population. Following Dickson and Stephens (2015), we focused on imitation rather than spontaneous gestures. Imitation of teachers' gestures could be a way to improve students' learning and to design new teaching methods. In the current study, we controlled the gestures the participants saw and made. We investigated the effect of imitating specific arm-hand gestures on the learning of structural and functional anatomy of the forearm. Our main hypothesis was that, as compared with just seeing the gestures made by someone else, imitating gestures during learning could improve the memorization of the structures' names (gestures as part of lexical representation and/or as cue to lexical access), structures' location (gestures as spatial cues) and/or the understanding of functions (gestures as support for mental imagery and conceptual learning). Indeed, a stronger activation of the motor system in the former case may create additional components to the memory traces and/or contribute to shape abilities to mentally simulate the function (Barsalou, 2008). The effect of making gestures was assessed by comparing two groups of participants (seeing vs. seeing and imitating gestures) both in terms of performance and subjective evaluation. All the evaluations were done after the learning session, either just after the session or a few days after, as previous work suggest that gestures “make learning last” (Cook et al., 2008). Gesture behaviors were also monitored during the learning and test phases and the quantity of gestures made was related to the scores in the different evaluations.

## Materials and methods

### Participants

Forty-five adult volunteers (aged 18–44, 27 females) gave written informed consent to participate in the study. They were recruited by advertisements and through a dedicated volunteers' database at Université Grenoble Alpes. Participants were informed of the inclusion criteria: French native speaking, no neurological or psychiatric disorders, normal or corrected to normal sight and hearing, novice knowledge of anatomy (to avoid ceiling effects and between-subjects differences in performance). All the selected participants had novice knowledge in general, functional and upper limbs anatomy (see Procedure section for details of participants' knowledge evaluation). All participants received a 15 € shopping gift card for their participation. The study was approved by the CERNI (Ethic Committee for Non Interventional Research) associated to the Université Grenoble Alpes.

### Distribution of participants into two comparable groups

Our experimental design required two groups of participants: a Gesture group (see and imitate the gestures made by the model) and a Control group (see the gestures made by the model). The distribution of participants into these two groups was done such as to balance various skills that could influence performances in anatomy learning (Hoyek et al., 2009; Berney et al., 2015). The following skills were evaluated during a pre-test phase:

- Visuo-spatial abilities, measured using a redrawn version of the Mental Rotation Test (MRT) from Vandenberg and Kuse (1978). We selected the first 12 items of the original test so that this pre-test was not too long for the participants (Peters et al., 1995).
- Mental imagery abilities, evaluated using a French translated version of the Movement Imagery Questionnaire-Revised (MIQ-R) from Hall and Martin (1997) introduced by Lorant and Nicolas (2004). This test consisted in eight questions in which participants had to evaluate their ability to mentally represent themselves doing a movement.
- Ability to memorize complex unknown words, assessed using a list of 8 pseudo-words with two or three syllables that we generated from the Lexique 2 database (New et al., 2001). The pseudo-words were selected to have different orthographic organization.

Participants were assigned to one of the two groups using profile pair matching according to their scores in the pre-tests. As far as possible, the age, gender, and educational level were also taken into account. Some of the participants firstly assigned to the Control group (*n* = 3) did not respect the instructions and made a significant number of gestures during the learning phase while they were not supposed to. They performed the entire study and received a gift card but were then replaced by three other participants such as to maintain the balance between groups. The final groups included 21 participants each, aged 25.6 ± 5.62 (mean ± standard deviation) in the Control group (13 females) and aged 27.7 ± 6.86 in the Gesture group (12 females).

### Learning and testing material

The educational material was a simplified lesson about the structural and functional anatomy involved in supination and pronation movements of the forearm, and associated tests of knowledge. As illustrated in Table 1, supination refers to an external rotation of the forearm and pronation to an internal rotation of the forearm (palm of the hand upwards and downwards at the end of the movement, respectively). This topic was chosen because the lesson could easily be simplified and participants' own forearms being within sight, they could be directly linked to the learning content. All the material was elaborated in collaboration with a professor of anatomy (the second author of this paper) in order to take into account both pedagogical and experimental objectives. The lesson and testing material were thus close to educational supports used in ecological situations of anatomy teaching while still respecting experimental constraints.

**Table 1:** Main steps of the lesson, associated diagrams, and gestures made by the model.

**Diagram**	**Gestures and content**	
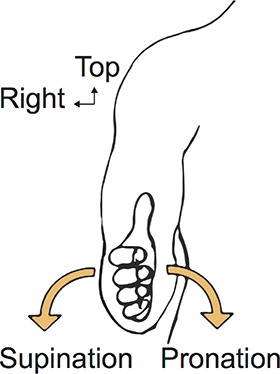	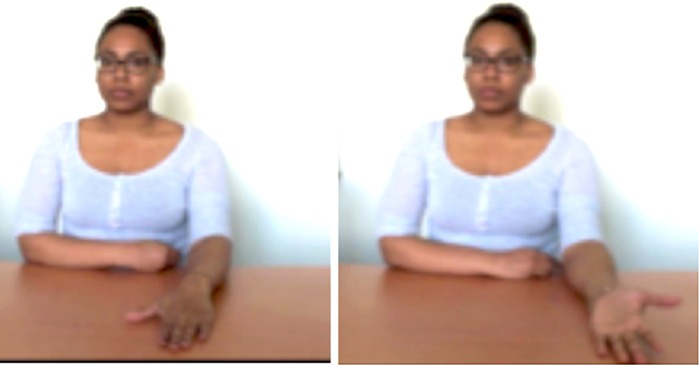	Step 1 (2 gestures, 1 slide). Supination and pronation movements of the forearm are introduced. The model makes pronation (left) and supination (right) rotating motions from the reference position (diagram on the left). Video shots display movements offset positions.
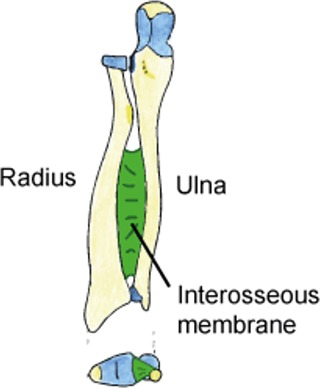	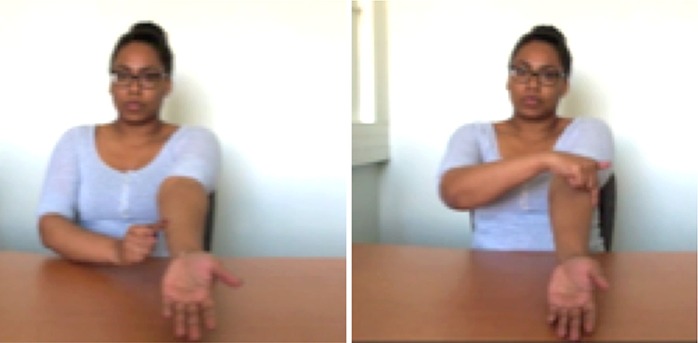	Step 2 (3 gestures, 4 slides). The structures of the forearm are explained (the original diagram includes more details presented in several steps, see Figure 1). Here, the model shows on her arm the location of the structures by moving her finger along her ulna (left) and her radius (right) bones. She then did the same for the interosseous membrane
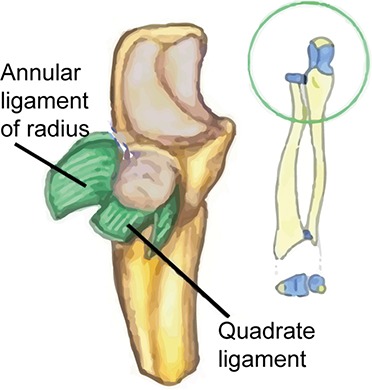	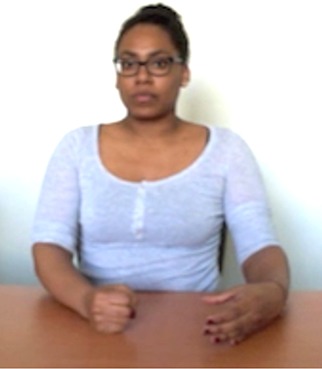	Step 3 (2 gestures, 1 slide). The main ligaments involved in supination and pronation and their function are explained. The model mimics the rotation of the radial head into the annular ligament of the ulna: she brings her right fist into her left hand and then executes rotational movements of her fist. A similar gesture was used for the second ligament: the quadrate ligament.
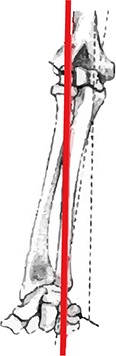	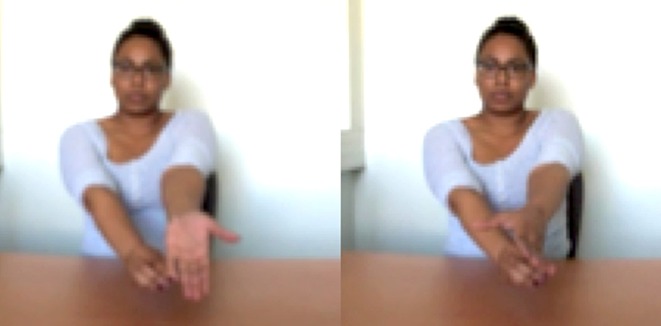	Step 4 (1 gesture, 1 slide). The rotation axis of pronation and supination is explained. The model shows the axis from the radial head to the top of her pinky finger. She then pivots her arm around this axis.
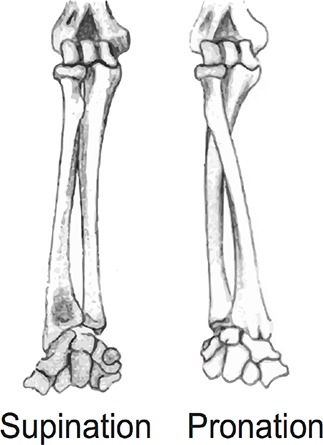	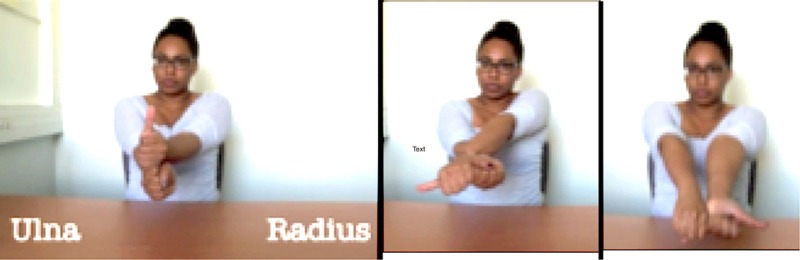	Step 5 (2 gestures, 1 slide). The function of the radius and ulna during pronation and supination are explained. The model associates one of her arms to the radius (thumb-up arm) and the other to the ulna. From left to right: in the neutral position (see diagram on row 1), the radius is on the top, it then pivots over the ulna in pronation while staying almost parallel to it in supination.

*The left column illustrates the principal diagrams displayed in the different slides. The right column provides a short description of the educational content and shots from the video illustrating the types of gestures made by the model (the model is the first author of this paper). Note that the model mirrors the diagrams. The number of gestures and slides are also specified for each step*.

#### Learning material

For the learning phase, a video was designed from educational slides that displayed the anatomical diagrams representing different parts of the forearm bones, joints, and ligaments. A soundtrack with explanations from the professor was synchronized with the slides. On the top-right of each slide, a video of the experimenter (the “model”) indicated the gestures to imitate or to observe. This video was always visible with the model staying still during audio explanations. An overview of the lesson is provided in Table 1. The lesson was 10 min long and included 8 slides and 10 gestures.

The gestures were inspired from those used by the professor in his real courses. They were mainly pointing-like and iconic-mime gestures (Kelly et al., 2008; Abner et al., 2015). They were designed such as to convey complementary information to the teacher's speech that should help understanding: the function (e.g., Step 1 in Table 1); the location of a structure in the forearm (e.g., Step 2 in Table 1); the structure itself (e.g., Step 3 in Table 1); or the implication of the structure in the function (e.g., Step 3–5 in Table 1).

At the end of each slide, the video showing the gestures was masked with a green square indicating to the participants that the gesture will be played again and that they would have to redo it (Gesture group) or to watch it again while staying still (Control group). The video was the same for the two groups except for the instructions written in the green screen.

#### Testing material

**Figure 1:**
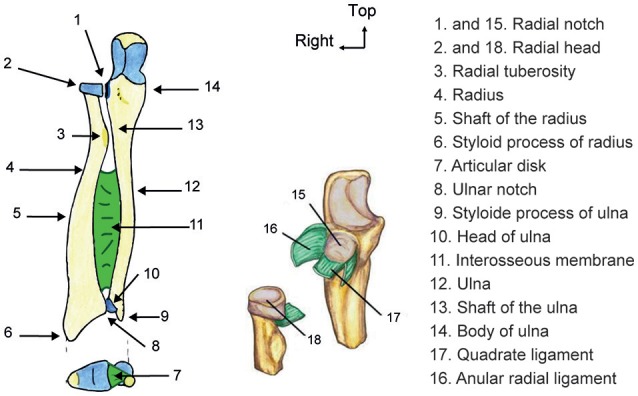
Main diagram of the forearm anatomy used in learning and testing phases. In total 14 different names had to be memorized on the main diagram (left) and two more on the diagram with ligaments (on the right). The two diagrams share two structures with different views (1 and 15; 2 and 18).

Learning of the lesson was assessed using four paper and pencil tests chosen to assess different levels of memory and knowledge:

- *Recall*: Participants write down on a white sheet, as many of the 16 structures names seen during the learning phase as possible (cf. the list of names on Figure 1).
- *Diagram*: Participants write down the names of the structures at the correct location indicated on the diagrams (cf. diagrams on Figure 1, without the numbers).
- *Diagram_Name:* Participants localize the names of the structures on the diagrams (cf. Figure 1). The diagrams were provided together with the list of the structures names in random order. Participants had to write the number corresponding to the correct location in front of the name of the appropriate structure.
- *Multiple Choice Questions (MCQ):* Participants had to choose the correct answer to six questions related to the understanding of functional aspects. For each question, participants circled one answer among four propositions (only one was correct).
- *Subjective evaluation:* Participants had to evaluate the clarity, motivation, interactivity, engagement of the lecture, and the usefulness of the gestures by evaluating their level of agreement with five assertions, one for each dimension (for instance, “I felt engaged during learning” to assess engagement). We used a Likert scale ranging from 1 (I strongly disagree, always the most negative judgment) to 10 (I fully agree, always the most positive judgment).

### Procedure

**Figure 2:**
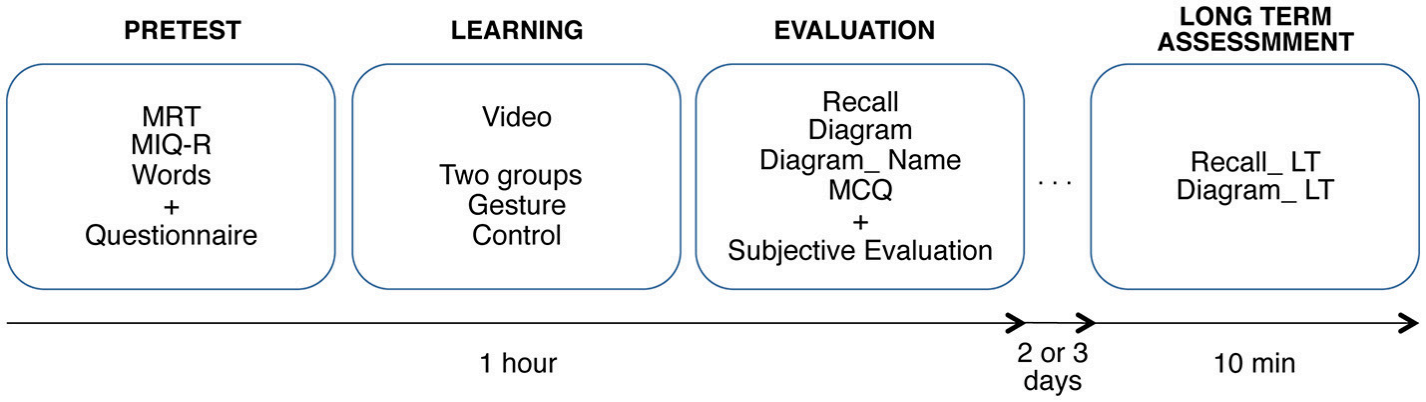
Overview of the procedure of the experiment (see text for details).

The experiment was about 1-h long. The participants were informed of the experimental conditions before starting the experiment. They filled a consent form and they gave their consent, or not, to be videotaped during the learning and test phases. The participants who agreed to be filmed (*n* = 33) signed an image-right form, which specified that the recordings will be used for the purpose of the study only. The procedure is summarized on Figure 2 and includes the following steps:

#### Pre-tests phase

The instructions for the two visuo-spatial tests were given and participants complete the MIQ-R and the MRT. They then filled a questionnaire to assess their previous knowledge in anatomy. They had to self-evaluate their level in general, functional and upper limbs anatomy by choosing between: no knowledge, beginner, intermediary, or expert. All of the selected participants indicated that they had no knowledge or a beginner level in anatomy, which we consider as being novice in the field. We also evaluated their knowledge relative to the content of the lesson (cf. complete the diagram displayed on Figure 1). Finally, they performed the word memory test. In this last test, the 8 pseudo-words were written on a sheet and the experimenter read them along with the participants. They had 1 min to memorize the pseudo-words and to retrieve them verbally. The experimenter wrote down the words recalled by the participants. Scores in MIQ-R, MRT, and words memory pre-tests were used to assign participants to each group: Gesture vs. Control (see above).

After the pre-tests phase, a digital camera was set-up to videotape the participants who agreed to be filmed. For participants who didn't agree, the experimenter took notes of the gestures made for each slide during the learning phase and during each test of the evaluation phase.

#### Learning phase

During the learning phase participants were seated at a table, in front of a 22-inch screen. All the participants were instructed to listen and to watch carefully the videotaped lesson displayed on the screen and to memorize as much information as possible. They were informed that a video of the experimenter would be played on the top right of the screen. In this video, the experimenter would make gestures that should help them to understand the content of the course. Participants in the Gesture group were instructed that they would have to reproduce the gestures after the instruction “it's your turn” would be displayed on a green square, and while the model performed the gestures again. Participants in the Control group were told to keep their hands on the table during the whole learning phase. Participants listened to the professor's explanations with headphones.

#### Evaluation phase

Right after the learning phase, participants completed the four tests of knowledge. They were informed that each test was time pressured (5 min each). The experimenter provided the tests to the participants in the following order: Recall, Diagram, Diagram_Name, and MCQ, on separated paper sheets and respecting the timing. Finally, participants completed the subjective evaluation.

At the end of the first session, the experimenter asked to the participants their agreement to call them back 2 or 3 days after the experiment to do a long-term assessment. When the participants agreed (19/21 participants in each group did), a paper-sheet with the diagrams displayed on Figure 1, was provided to them (without the list of names). They were instructed not to revise anything about the lesson before the call, and to wait for further instructions.

#### Long-term assessment

The long-term assessment was performed by phone and lasted about 10 min. Phone call was chosen to reduce the loss of participants and preserve the balance between groups.

During the phone call, the experimenter informed the participants that they would have to perform the Recall and Diagram tests again, with time pressure as well. First, she asked the participants to recall as many as possible of the structures studied during the learning (Long-Term Recall). She then asked the participants to watch the diagrams and to give the name of the structure corresponding to each number (Long-Term Diagram). The experimenter wrote down participants' answers, including mispronunciations.

### Scoring in the different evaluations

#### Tests of knowledge

Scoring was crosschecked by the first and the last author. The scores were expressed as a proportion of correct answers relative to the total number of items.

- *Recall scoring (short and long term)*: 16 names of structures were expected. Each provided item was scored as 1 if identical to one of the expected name, or a synonym/equivalent name also used by the professor during learning (cf. head of the radius for radial head).
- *Diagram test scoring (short and long term)*: An item was rated 1 if the name of the structure was exact and if it was correctly located on the diagrams.
- *Diagram_Name scoring*: Each structure correctly localized on the diagrams was rated 1.
- *Multiple Choices Questionnaire (MCQ):* One point was given for each correct answer.

For Recall and Diagram, we also computed the proportion of common letters between the expected and recalled words as it was done in a recent study using a similar assessment test (Skulmowski et al., 2016). We chose to use the orthographic distance of Levenshtein, an indicator of spelling proximity (Levenshtein, 1966). This more complex analysis led to the same conclusions as the “all-or-nothing” scoring, so it is not reported here.

#### Subjective evaluations

For the subjective evaluations, a score out of 10 was obtained for each dimension and participant.

#### Gesture behaviors

Gestures made by participants were counted using the ELAN software (version 4.9.2, Lausberg and Sloetjes, 2009) if a videotape was available, or using the experimenter's observations otherwise. For each participant we obtained a number of gestures for each step of the learning phase and then for each test of day 1 (Recall, Diagram, Diagram_Name and MCQ).

### Experimental design, hypotheses, and statistics

The main response variables were:

- The proportion of correct responses in the different tests (PROP_CORRECT, values from 0 to 1);
- The five subjective evaluations of the lecture (QUAL_EVAL, considered as an ordered variable with values from 1 to 10).

The main factors tested were:

- GROUP (Control group vs. Gesture group), between-subject factor;
- MOMENT (Short-term vs. Long-term), within-subject factor;
- TEST_TYPE (Recall vs. Recall_Diagram vs. Diagram vs. Diagram_Name vs. MCQ) within-subject factor;
- DIMENSION (Clarity, Motivation, Interactivity, Engagement, and Usefulness), for the subjective evaluation only.

The following hypotheses were tested using the version 3.2.2 of the R software (R Core Team, 2015; R Development Core Team, 2016):

#### A. making gestures may improve learning; the effect should last in long-term evaluations

We were expecting a significant effect of GROUP, with greater performances in the Gesture than in the Control group on PROP_CORRECT. This effect could vary depending on TYPE_TEST and on MOMENT with two main expectations: (1) if imitating gesture improves the mental simulation of the movement it should be particularly beneficial for the MCQ test that evaluates functional anatomy (significant GROUP ^*^ TYPE_TEST interaction); (2) if imitating gesture helps consolidating memories more than just seeing gestures, between groups difference should be greater in the long-term evaluation than in the short-term one (significant GROUP ^*^ MOMENT interaction).

As PROP_CORRECT ranged between 0 and 1, it was analyzed with a Beta regression, a type of multiple linear regression, using the “glmmADMB” package (version 0.8.3.3, Bolker et al., 2012). A correction was applied to the data to get values in [0;1] range, as required by the method (Smithson and Verkuilen, 2006). The contribution of each variable was evaluated using a step-by-step descendant procedure with a backward method: we started the analysis with the full model (containing all the variables and all the interactions) and then step-by-step, we suppressed the terms that were not critical to the model. This was done by comparing models with and without the variable of interest using likelihood ratio tests (the *anova* function of the package *stats*, 2016.3.3.2 in R). Differences between models were considered significant for *p* < 0.05. PARTICIPANT was included as a random factor.

As Diagram_Name and MCQ were not available for the long-term assessment, it was not possible to include these two tests in the model evaluating the effect of MOMENT. Therefore, we ran two different models:

- Model_ST (short-term): effect of GROUP and TYPE_TEST for short-term data only, all the participants, starting with:
  $$\begin{array}{l} PROP\_CORRECT\sim GROUP{\;}^{*}\;TEST\_TYPE\\ \;+(1|PARTICIPANT) \end{array}$$
- Model_LT (short-term and long-term): effect of GROUP, TYPE_TEST_LT (Recall and Diagram) and MOMENT and participants with long-term data only (PARTICIPANT_LT, *n* = 19 in each group):

$$\begin{array}{l} PROP\_CORRECT\sim GROUP{\;}^{*}\;TEST\_TYPE\_LT{\;}^{*}\;MOMENT\\ \;+(1|PARTICIPANT\_LT) \end{array}$$

The selected models were then used to run multiple comparisons based on contrast matrixes, using the “multcomp” package (version 1.4-6) and the function “glht” in R (Hothorn et al., 2008). This package applied corrections for multiple comparisons.

#### B. making gestures may improve the subjective evaluation of the lecture

We were expecting that moving their body during the learning phase could improve the way participants evaluated the clarity, motivation, interactivity, engagement, and gestures usefulness dimensions of the lecture. The effect of making gestures on QUAL_EVAL according to the DIMENSION was evaluated using an ordinal regression (function clmm of the package Ordinal in R 2015.6-28, Christensen, 2015). The principle is similar to the Beta regression with a full model at the beginning and a backward step-by-step method. The full initial model was the following:

$$QUAL\_EVAL\sim GROUP{\;}^{*}\;DIMENSION+(1|PARTICIPANT)$$

#### C. the quantity of gestures made during learning and testing phase may influence the performances

We finally analyzed the number of gestures made during the different phases and when possible, correlated this number to scores in the different evaluations.

## Results

### Constitution of groups

We first controlled that the two groups were balanced in terms of pre-test knowledge, MRT, MIQ, words memory abilities, and other dimensions that could affect anatomy learning.

#### Initial knowledge of anatomy

Only two participants correctly locate the radius on the diagram filled before learning and none of the participants evaluated themselves as being experts in functional anatomy and anatomy of the upper limbs. All the participants are thus considered to be novices in regards to the content of the lesson.

#### Balance between groups

Table 2 provides, for each group, the average values in each of the tests used to balance groups (MRT, MIQ, Words), as well as the average age, educational level and number of days between short-term and long-term evaluations. Mann-Whitney non-parametric tests were used to compare the two groups on each of these six dimensions. None of the differences between groups are significant (without correction, in all cases: *W* < 217, *p* > 0.22), suggesting that the two groups are globally comparable on these dimensions. This was also the case after removing the four participants that didn't perform the long-term assessment.

**Table 2:** Group description in terms of visuo-spatial abilities (MRT, MIQ), memory (Words), age, educational level, and number of days between learning and long-term assessment.

	**Control (*n* = 21) Mean ±SE**	**Gesture (*n* = 21) Mean ±SE**
MRT	9.1 ± 0.69	8.9 ± 0.82
MIQ	42.3 ± 1.26	43.9 ± 1.23
Words	4.8 ± 0.36	4.8 ± 0.34
Age (years)	25.6 ± 1.23	27.7 ± 1.50
Educational Level (Nb. of years of studies after high school diploma)	3.1 ± 0.48	3.5 ± 0.51
Nb. of days	2.4 ± 0.18	2.6 ± 0.15

### Effect of GROUP and TYPE_TEST in the short-term session

**Figure 3:**
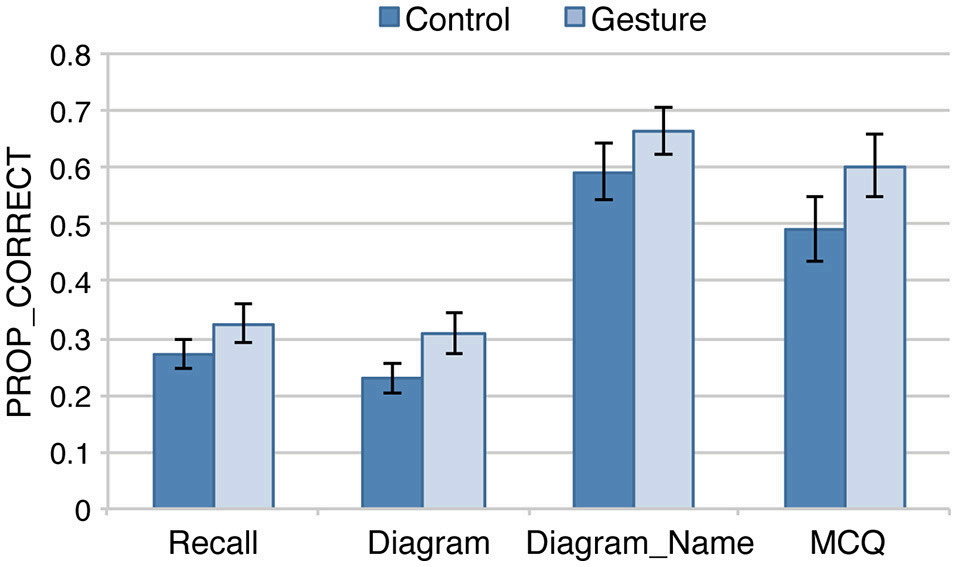
Average proportion of correct responses (PROP_CORRECT) in each test and group in the short-term session (*n* = 21 in each group). Error-bars represent between participants standard errors.

The average performances in the short-term session are displayed on Figure 3. Regardless of TEST_TYPE, average scores are always greater in the Gesture than in the Control group. Greater performances are also observed in Diagram_Name and MCQ as compared with Recall and Diagram.

The beta regression analysis (Model_ST) shows that the contribution of GROUP ^*^ TEST_TYPE is not significant [$\chi_{\left(3\right)}^{2}$ = 3.9, *p* = 0.27], nor the global effect of GROUP [$\chi_{\left(1\right)}^{2}$ = 1.9, *p* = 0.17]. By contrast, TYPE_TEST significantly improves the model [$\chi_{\left(3\right)}^{2}$ = 111.3, *p* < 0.001], with greater scores in MCQ and Diagram_Name than in Recall and Diagram (for all paired comparisons between the two groups of tests: *z* > 5.9, *p* < 0.001). It is not surprising that participants performed better in the Diagram_Name than in the Recall and Diagram tests, as the structures names are provided in Diagram_Name. Comparison with MCQ is less relevant as the level of difficulty is not standardized between this test and the others. The important point here is that TEST_TYPE effect doesn't significantly depend on the group.

### Effects of GROUP, TYPE_TEST_LT, and moment

**Figure 4:**
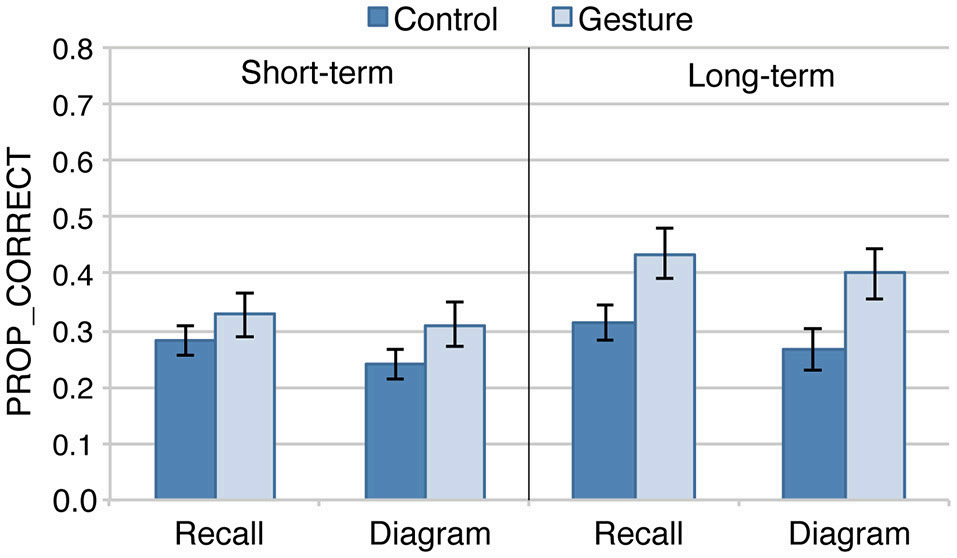
Average proportion of correct responses (PROP_CORRECT) for the participants and the tests performed in both the short-term and long-term session (*n* = 19 in each group). Error-bars represent between participants standard errors.

Figure 4 displays the average results for each GROUP and MOMENT when analyses are limited to TYPE_TEST_LT and PARTICIPANT_LT. Short-term results are consistent with those observed when considering all the participants for the Recall and Diagram tests. Long-term results suggest however an increase of the differences between the two groups in the long-term session, and suggests that long-term improvements were mostly limited to the Gesture group.

The beta regression analysis (model_LT) shows no significant contribution of the three-levels interaction [GROUP ^*^ TYPE_TEST_LT ^*^ MOMENT, $\chi_{\left(1\right)}^{2}$ = 0.01, *p* = 0.91], nor of TYPE_TEST_LT ^*^ MOMENT [$\chi_{\left(1\right)}^{2}$ = 0.44, *p* = 0.51], nor of GROUP ^*^ TYPE_TEST_LT [$\chi_{\left(1\right)}^{2}$ = 1.0, *p* = 0.31]. By contrast, TYPE_TEST_LT significantly contributes to the model, with greater performances in Recall than Diagram test [$\chi_{\left(1\right)}^{2}$ = 8.7, *p* < 0.01], as well as the GROUP ^*^ MOMENT interaction [$\chi_{\left(1\right)}^{2}$ = 8.3, *p* < 0.01]. Contrast comparisons were then performed on this last interaction. They show no significant difference between groups for short-term assessments (*z* = 1.22; *p* = 0.29), but significant difference for long-term assessments (*z* = 2.36; *p* < 0.03). Supplementary analysis suggests that the improvement at long-term was significant for the Gesture group (z = 3.26, *p* < 0.01) but not for the Control group (*z* = 1.38, *p* = 0.28)

### Subjective evaluation

**Figure 5:**
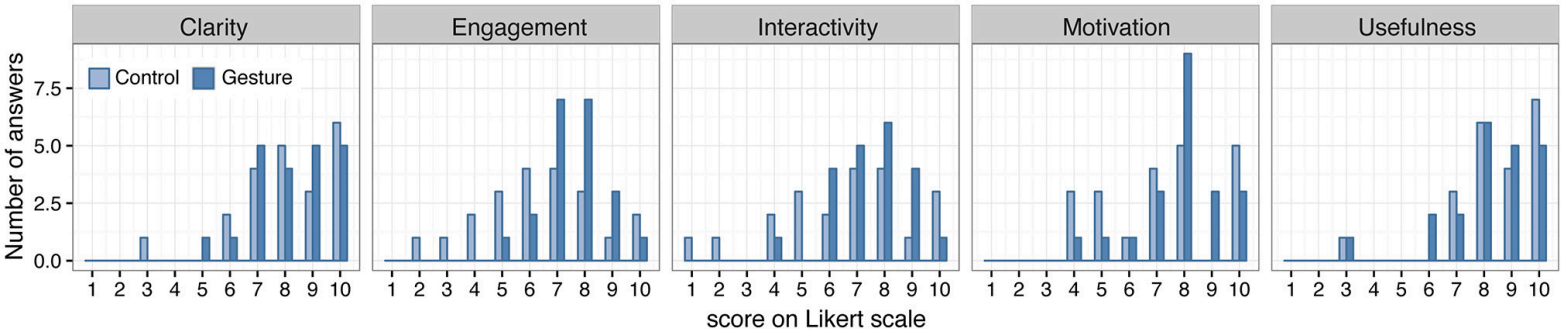
Distribution of QUAL_EVAL scores (subjective evaluations on Likert-scale, 10: most positive evaluation) for each DIMENSION depending on the GROUP.

Distributions of subjective evaluation scores (QUAL_EVAL) are displayed on Figure 5. QUAL_EVAL is distributed in an equivalent way in the two groups for the clarity of the lecture and the usefulness of the gestures. For the other dimensions (engagement and motivation in the lecture, interactivity of the lecture), participants in the Gesture group seem to provide higher scores than participants in the Control group.

The ordinal regression shows no significant contribution of GROUP ^*^ DIMENSION interaction [Likelihood ratio statistic: *LR.stat*_(4)_ = 6.7, *p* = 0.15], nor of GROUP [*LR.stat*_(1)_ = 1.12, *p* = 0.29]. By contrast, QUAL_EVAL significantly depends on DIMENSION [*LR.stat*_(4)_ = 34.25, *p* < 0.001], with more positive evaluation in general of Clarity and Usefulness than Motivation, Interactivity, and Engagement. Note that when dimensions are compared independently using Mann–Whitney tests, Engagement is significantly better evaluated by the Gesture than the Control group (*W* = 135.5, *p* = 0.03), while there is no significant between-groups differences for the other dimensions (*W* > 171.5, *p* > 0.2). This supplementary analysis was run for comparison purposes with a previous work based on mean comparisons, suggesting that students feel more involved in the lecture when making gesture than when they don't (Oh et al., 2011).

### Gestures analysis

#### Gestures made during learning

**Figure 6:**
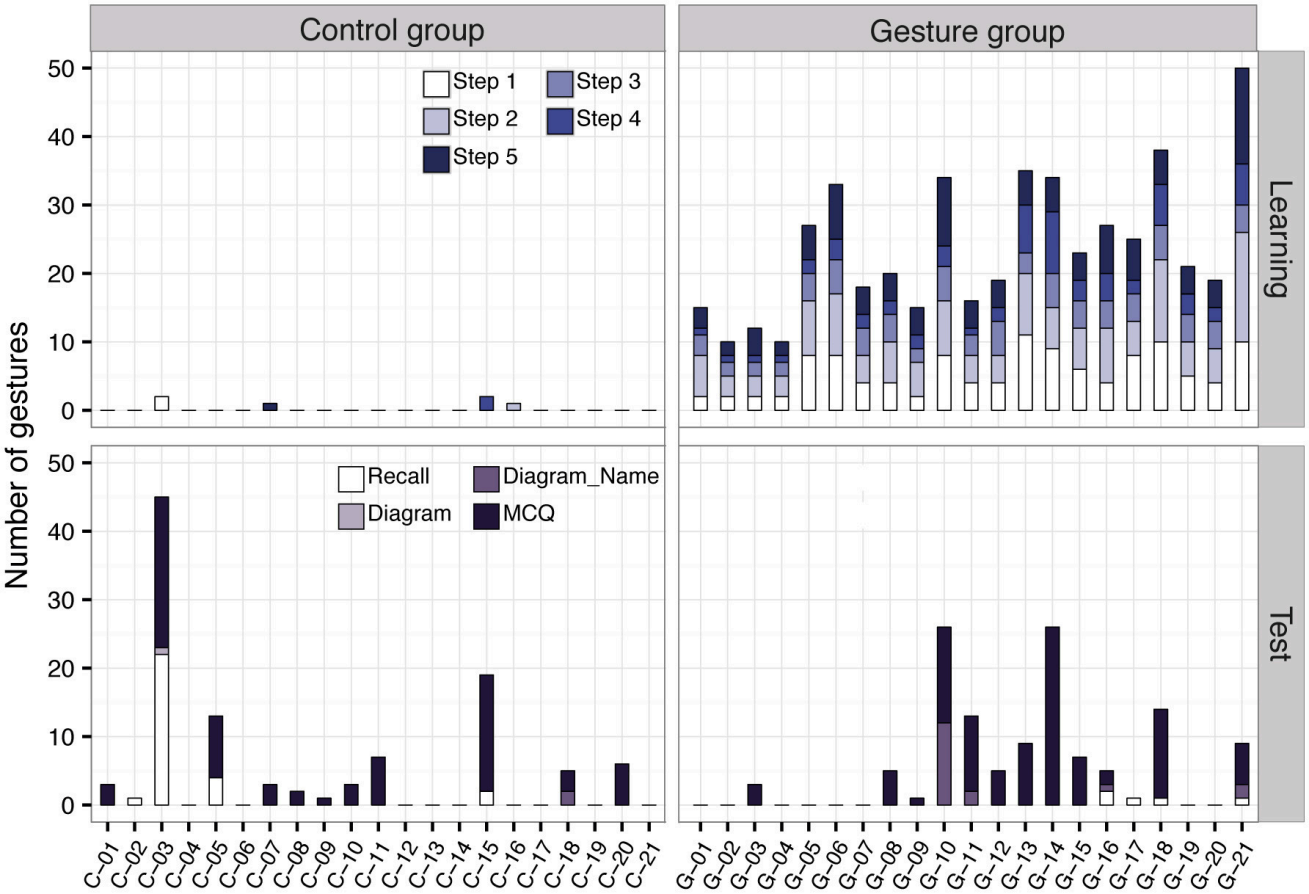
Number of gestures made by each participant during the learning phase (first row) and the evaluation phase (second row). Results are given for each step of the learning phase and each test in the evaluation phase. The number of expected gestures was: 2 gestures in Steps 1, 3 and 5; 3 gestures in Step 2; 1 gesture in Step 4, see Table 1 for details.

As described in the Materials and Methods section (cf. Table 1), the model made 10 gestures during the learning phase. We analyze to which extent the participants reproduced these gestures. The majority of the participants in the Control group stayed still during learning but few of them (*N* = 4) made 1 or 2 gestures (Figure 6, top left), at variable steps of the lesson. We still included them in the control group, as these gestures were occasional. In the Gesture group, all of the participants made the 10 expected gestures at least, but most of them repeated the gestures several time, with more than half of the participants making at least 21 gestures in total (Figure 6, top right). As an illustration of specific behaviors, the participants G-02 and G-04 made each gesture once, while G-21 repeated each gesture several times, up to 50 gestures in total.

#### Gestures made during the evaluation phase

As displayed on Figure 6, second row, during the evaluation phase, participants' behaviors were variable and depended on the test. Globally, the participants poorly gestured when completing Recall (number of participants making at least one gesture: *n* = 4 both in Control and Gesture group), Diagram (*n* = 1 in Control, 0 in Gesture) and Diagram_Name (*n* = 1 in Control, *n* = 4 in Control). Outlier behaviors are also observed. For example, C-03 and G-10 made 22 and 12 gestures when completing Recall and Diagram_Name respectively. More gestures were made during MCQ, with about half of the participants in each group (*n* = 11 in Control and *n* = 12 in Gesture) who produced at least one gesture when completing this test.

#### Relationship between gesture behavior and evaluation results

Finally, we tested if the gesture behaviors during the learning and then the test phases were related to the results in each evaluation using Spearman correlations. In the Gesture group, no reliable correlation is observed between the number of gestures made during the learning phase and PROP_CORRECT in Recall, Diagram, Diagram_Name, and QCM, both for short-term and long-term evaluations (*r-*values range from 0.09 in Recall to 0.27 in MCQ). This is also the case for QUAL_EVAL, regardless of the dimension (*r-*values range from −0.38 for Engagement to 0.02 for Clarity). Because about half of the participants didn't make any gestures during the different evaluation tests, it is not possible to reliably correlate the number of gestures made to the scores in the different tests.

## Discussion

The purpose of this study was to provide further evidences that making gestures can improve the acquisition of new knowledge. Anatomy learning was chosen as a specific case of embodied learning and as a challenging topic to learn. In particular, we focused on the specific contribution of imitating gestures in relation to the learning content as compared with just seeing them. Indeed, a stronger activation of the motor system in the former case could be more beneficial to the reenactment processes supposedly supporting the cognitive abilities (embodied cognition, see Barsalou, 2008). To this end, we compared performances in learning anatomy of the forearm between two groups of novice learners: a Gesture group who reproduced the gestures made by a model during learning and a Control group who only saw these gestures. Overall, the Gesture group appeared to perform better than the Control group but the differences were significant only in the long-term subset of evaluations. Short-term subjective evaluations suggest that the Gesture group felt more engaged in the lecture than the Control group. We also observed a greater tendency to make gestures during MCQ functional evaluation than recall or structural assessments, with yet large between-participants variability. These results are discussed in respect to previous empirical and theoretical work, experimental limits, and new perspectives.

### Making/imitating gestures vs. seeing gestures

Our study investigated the effect of the implication of the motor system when learning structural and functional anatomy. The results suggest that “imitating gestures” is more effective than just “seeing gestures” while learning the names of anatomical structures. The beneficial effect of doing gestures was previously observed at short-term in a mental rotation task (Goldin-Meadow et al., 2012) and is consistent with the idea that “motor information accrued by the body can affect learning and development by grounding mental representations in motor areas of the cortex and structuring associated perception” (Kontra et al., 2012, p. 732).

In most of the previous works about the role of gestures in learning, “making gestures” usually overlap “seeing gestures,” meaning that the participants who don't reproduce the gestures don't see them neither (Cook and Goldin-Meadow, 2006; Cook et al., 2008). Previous works have highlighted that seeing the teacher's gestures is helpful for learning (Cook et al., 2013). The impact of being active during learning is an important issue in the research about learning and memory.

In the current study, imitation was chosen rather than spontaneous gestures to limit differences between the participants during the learning phase and to contrast seeing vs. making gestures with equivalent gestures in the two conditions. It could yet be interesting to compare the effects of imitating the teacher's gestures vs. making spontaneous gestures on learning anatomy. Further work should evaluate the interaction between the type of gestures made by the participants and the kind of information they have to memorize. Our results still suggest that teachers making gestures and encouraging students to imitate them during a lecture could have positive effects on learning.

Numerous studies have shown that being active during learning lead to better recognition (Liu et al., 2007; Meijer and Van der Lubbe, 2011) or faster recognition (Harman et al., 1999; James et al., 2001). This effect is observed in different tasks such as matching of objects (Sasaoka et al., 2010), textures discrimination (Lee and Wallraven, 2013) or recognition of action sentences (Hornstein and Mulligan, 2004). The current study provides supplementary evidence in this direction. One could argue that in our study, imitating gestures may have increased participants' attention to the lecture and/or the gestures, leading to a better representation of the content of the lecture and of the associated gestures. Evidence suggesting that action itself rather than attention supports active learning is provided by Meijer and Van der Lubbe (2011): an active exploration of 3D objects leads to a better recognition of these objects than a passive exploration, even when participants had to carry out a secondary task during learning.

Previous work also suggests that being active during the learning of anatomy improve the feeling of involvement, especially when innovative methods are used (cf. Sugand et al., 2010). In our study, participants who made gestures tended to evaluate the lecture as more engaging than the participants who did not make gestures. There was no further significant difference for the other qualitative evaluations (clarity, motivation, interactivity, usefulness of gestures). It is possible that the chosen questions were too broad to catch differences between groups or that “seeing gestures” is indeed equivalent to “imitating gesture” at a conscious level. It is also possible that naïve participants are not able to distinguish the impact of the gestures, as they never attended an anatomy lecture. Comparisons with students in medical school or physiotherapy are required to assess this point.

An original aspect of our approach was also to report the number of gestures spontaneously made by the participants during the short-term tests. The quantity of gestures made was limited overall and was clearly participant-specific. Some of the participants might be uncomfortable with making gestures especially when videotaped or observed by someone else, as it was the case in the current study. It will be interesting to run specific studies to test the interaction between experimental conditions and participants' profiles (gesturers vs. non-gesturers). In addition, our analyses suggest that about half of the participants spontaneously gestured when performing the MCQ test. This test is closer to problem-solving situations, requires functional dynamic knowledge, and probably relies more on visuo-spatial and body representations than recall and diagram tests. Further work should analyze the effect of gestures on performance in functional vs. structural anatomy tests. Investigations of brain activity during testing phase would also allow more direct assessment of the motor system involvement in the different evaluations: some participants in the Gesture group may not gesture but their motor system might still be more activated than that of the participants in the Control group.

### Short-term vs. long term performances

An important result of this study is the significant effect of making gestures on performance in long-term assessment only. Furthermore, the participants in the Gesture group were not only better than those of the Control group: they also significantly improved their scores as compared with short-term assessment. Similar beneficial effects of gestures on long-term learning was previously observed in several studies when the post-test occurred 2 days after learning (Hornstein and Mulligan, 2004) or even 3–4 weeks after learning (Cook et al., 2008, 2010). These results can be related to the effects of sleep on memory. Literature broadly reported some positive effects of sleep on memory consolidation (Diekelmann and Born, 2010; Rasch and Born, 2013). During sleep, the neuronal networks involved in learning are reactivated (Wilson and McNaughton, 1994; Ji and Wilson, 2007) and there is a dialogue between hippocampus and neocortex that consolidates memories (Buzsáki, 1996; Landmann et al., 2014). Therefore, 1 or 2 nights of sleep could enhance memory traces by a (re)-simulation of experience (reactivation in the hippocampus) and a reorganization of the memory traces (by the dialogue between hippocampus and neocortex).

The stronger motor activation when imitating gestures as compared to seeing gestures during learning may influence the reactivation of experience and the reorganization of memory during sleep, favoring the integration of the motor component. Sleep has a beneficial effect both on declarative and procedural memory (Plihal and Born, 1997; Rasch and Born, 2013). For example, sleep is well-known to have a positive impact on procedural memory and visuo-spatial tasks (Peigneux et al., 2004; Fischer et al., 2006; Rasch et al., 2007), as well as in motor performance (Huber et al., 2004). The difference between the two groups in the current study also suggests that “seeing gestures” vs. “imitating gestures” may create different memory traces. Future works should also assess the interaction between imitation vs. spontaneous gestures with the long-term integration of the motor component. Indeed, in the first case, participants see and provide a specific effort to imitate the teacher, while in the second case, they create their own motor path toward knowledge.

We were expecting beneficial effects of imitating gestures at short-term. We also added the long-term assessment in relation to previous work showing long-term effect of gestures on learning (Cook et al., 2008). For practical reasons, the long-term assessment was performed by phone. This limited testing possibilities but reduced the loss of participants, which was important to preserve balance between groups. Both groups went through the same procedure: there was no reason to suppose that participants in one group would respect differently the instructions than those in the other group. Regardless of these limits, we can make two important points relative to short-term vs. long-term assessment. (1) the positive effect of imitating gestures could be present at short-term but difficult to observe due to between-subjects design and to the multi-dimensional competences involved in complex learning such as anatomy. Between-subjects design should anticipate large groups of participants to compensate high between-subjects variability related to complex learning. (2) longitudinal assessments should be planned systematically in learning studies, especially when comparing learning conditions, as some conditions may need sleep and/or time to be integrated. Our study represents a first step in that way, showing that involving the body can be useful to keep information in mind as long as possible.

### Structures with gesture vs. without gesture

In our study, some of the learned structures were not associated with any gesture (see diagram on Figure 1 and Table 1). We ran some *post-hoc* analyses to compare performances on structures with vs. without gesture by computing two scores by participant: the proportion of correct answers for the five structures with gesture and for the 11 structures without gesture. Scores for the condition “without gesture” were much lower than those in the condition “with gesture.” However, there were more structures without gesture than with gesture and more importantly, structures without gesture were more complex in terms of vocabulary and were mostly subparts of the structures with gesture. The important point of this *post-hoc* analysis is that the effects of CONDITION (making gestures vs. seeing gestures) and MOMENT (short-term vs. long-term) were observed for structures with and without gesture. This result suggests that imitating gestures may help memorizing associated knowledge, provided without gesture.

### Why/how doing gestures could improve long-term performances in recall/diagram in an embodied cognition perspective?

In terms of cognitive processes, we can assume that doing gestures during learning is a way to enhance the memory traces associated. According to embodied cognition theories (Wilson, 2002; Barsalou, 2008), cognition emerges in the interaction between the body, the mind and the environment. In this framework, multiple traces models of long-term memory can provide some explanation of the effect of making gestures during learning. In these models, memory is defined by every sensory and motor component activated while experiencing the events from daily life (Logan, 1988; Versace et al., 2014). Several memory models are developed according to this idea and usually describe that the number of components of a memory trace can influence its strength (Versace et al., 2014). In this way, we can consider that having a supplemental motor component during learning (for the Gesture group) can strengthen the memory trace associated to this learning, if there is enough time (and/or sleeping processing) between learning and retrieval. Consequently, this memory trace is more distinctive than the others and will be retrieved easier. The embodied cognition theories also suggest that memory is a simulation of previous experiences (Barsalou et al., 2005; Barsalou, 2009). This simulation process is supposed to subtend different cognitive activities, including perception, memory, conceptual processing, as well as language comprehension and social cognition (see Barsalou, 2008). The implication of body states in memory traces created during the learning of anatomy should be addressed more directly, for example, using neurophysiological recording during the evaluation phases.

### Gesture type and learning content

Anatomy of the arm was chosen as topic as a specific case of embodied learning: gestures involved in learning were directly related to the learning content and participants could represent the concepts and vocabulary in relation to their own body. Moreover, the upper limb is visible and this information can be used as a clue for retrieval, which is not the case for anatomy of the face for example (Dickson and Stephens, 2015). An opened question is then: is the beneficial effect of gestures related to the type of link between the gestures and the knowledge to learn? Would the benefit from gestures be equivalent for another topic, unrelated to the body? Would less specific gestures (e.g., abstract gestures) lead to the same effects? Gestures seem to be useful in different domains (McCafferty, 2004; Goldin-Meadow and Alibali, 2013; Trofatter et al., 2014; Novack and Goldin-Meadow, 2015) but the relevance of the link between learning content and gestures type remains an opened question. In our study, participants who gestured during testing mainly did so during MCQ functional test. Understanding the embodiment of learning through gesturing will require investigating more specifically the link between the gestures types and the nature of learning itself. Anatomy learning could be used to manipulate this link and provide a major contribution for embodiment theories and educational approaches. Our study was a first step in that direction.

## Ethics statement

This study was carried out in accordance with the recommendations of Ethic Committee for Non Interventional Research (CERNI, Grenoble Alpes University) with written informed consent from all subjects. All subjects gave written informed consent in accordance with the Declaration of Helsinki. The protocol was approved by the CERNI.

## Author contributions

MC, AR, JT, and OP: Designed the experiment, interpreted the results. MC and OP: Created the material for the experiment. MC: Performed the experiment. MC, AR, and SG: Analyzed the data. MC and AR: Wrote the manuscript. JT, OP, and SG: Performed revisions and approved for the manuscript for publication.

### Conflict of interest statement

The authors declare that the research was conducted in the absence of any commercial or financial relationships that could be construed as a potential conflict of interest.
